# Communications

**Published:** 1910-11

**Authors:** Frederick S. Lee

**Affiliations:** Columbia University


### COMMUNICATIONS

### AN OPEN LETTER CONTRIBUTED TO THE NEW YORK TIMES BY PROFESSOR FREDERIC S. LEE PROFESSOR OF PHYSIOLOGY IN COLUMBIA UNIVERSITY, REGARDING THE ANTI-VIVISECTION EXHIBIT OF THE NEW YORK ANTI-VIVISECTION SOCIETY.

*To the Editor of The New York Times*:

There has been held in this city during the past ten weeks a public exhibition purporting to demonstrate the methods that are employed in laboratories of animal experimentation. It is held under the auspices of the New York Anti-Vivisection Society, an organization which for the past two years has endeavored in various ways to keep itself in the public eye. The exhibition has attracted less attention from the public than it deserves, for, while its scientific character may be questioned, it is valuable as affording a clue to the moral character of an organization which lays claim to a position of moral leadership. There are some of us who have entertained grave doubts as to whether this claim is justified, and these doubts have increased as the various successive acts of the society have been performed since the day of its birth. A study of its exhibition tends to increase these doubts.

The most graphic feature of the exhibition is an array of stuffed animals, some attached to operating tables, some with heads attached to surgical head-holders, some in partial dissection, with surgical instruments lying about, and in one case with a pool of red liquid, stimulating blood. The good taste manifested in the public showing of such gruesome sights may well be questioned and especially in view of the fact that a considerable number of the visitors which one sees at the exhibition are children. They are not only welcomed and allowed to roam freely about the room, but the unpleasant details of the exhibit are explained to them by the women attendants in charge, and a morbid curiosity is thus encouraged. The walls bear many pictures of animals, some undergoing luridly red surgical operations, some exhibiting anatomical dissections, and others participating in a variety of scenes of happiness or misery. The investigator is now and then shown, with a face of diabolical glee, gloating over his victim. A considerable number of portraits of men are shown, chiefly literary men and clergymen, with extracts from their writings, expressing more or less opposition to animal experimentation. In many cases these expressions are direct responses to requests by member of the society, and their language shows the degrading influence of the literature circulated by the society.

A significant part of the exhibition consists of the tales that are told to the visitors by the women attendants. Of the various operations that are portrayed or suggested, one is frequently told that they are customarily performed without anaesthetics, a statement which is not true. One attendant said to a visitor that the surgical head-holders were used for the purpose of breaking the jaws of dogs, and that this was done without anaesthetics. When questioned as to the reason for breaking the jaws of dogs she confessed ignorance. Such a procedure is so patently fantastis as to render comment unnecessary. There is an oven, heated by gas burners which contains the stuffed body of a rabbit, and which the attendant tells you is used for the purpose of baking live animals to death, and this also is performed without anaesthetics. Fabrication and grotesqueness here reach their culmination, for the oven is an apparatus intended for the incineration of dead organic matter, the automical refuse of a laboratory! The attendants are ever ready to discuss animal experimentation, seemingly quite unaware of their great and prejudiced ignorance. They do not hesitate to speak of well-known and highly-respected scientific men in intemperate language that is anything but refined or parliamentary.

To one who is familiar with laboratory procedure, the keynote of this exhibitin is falsity. The visiting layman can hardly fail to carry away with him a wholly incorrect notion of what animal experimentation means, what its methods are, and what a measureless amount of good it has accomplished for both the human race and the lower animals. Nowhere is there a sincere desire for the truth; everywhere there is ignorance, misrepresentation and false implication; everywhere the calmness of balanced judgment is wanting; everywhere there is an unbridled appeal to sentiment and to sentiment inflamed into passion. The ar mis great that is allowed to spread uncheck through a community the harm that may thus be done to the individual, but when such an influence is that may be done to the multitude is incalculable. Such an influence is both intellectually and morally debasing. When a bishop of the Christian Church, innocent of the truth and moved only by a blind rage excited by the misleading tales of this society, writes of the beneficent method of animal experimentation, a method from which he and his followers unwittingly derive daily blessings. "I have long been an enemy to vivisection, and am so still, * * * I would like to see it totally abolished and made an offense against the law * * *I am heartily in sympathy with the effort, not only to reform, but to destroy and root out altogether this sin against the lives of innocent creatures," we may well ask whether the time has not come for enlightened people to band together in opposition to this variety of fatuous fanaticism?

In the exhibition of which I write the most striking single exhibit is the New York Anti-Vivisection Society itself. It has had every opportunity to learn the truth or the falsity of its demonstration and its declarations. It has been told by those who know how untrue they are, and yet it has continued week after week to keep its deceptive sights before the public and to tell its false tales. In the minds of those who both know and respect the truth the New York Anti-Vivisection Society stands, under the deceitful mask of a pretended moral leader, as an obscurantist, a partisan of vicious principles and practices, and a foe of the public good.

FREDERICK S. LEE.

Columbia University, Feb. 4, 1910.

